# Intake of Dairy Products in Relation to Periodontitis in Older Danish Adults

**DOI:** 10.3390/nu4091219

**Published:** 2012-09-04

**Authors:** Amanda R. A. Adegboye, Lisa B. Christensen, Poul Holm-Pedersen, Kirsten Avlund, Barbara J. Boucher, Berit L. Heitmann

**Affiliations:** 1 Research Unit for Dietary Studies, Institute of Preventive Medicine, Frederiksberg Hospital, Copenhagen, DK-2000, Denmark; Email: blh@ipm.regionh.dk; 2 Department for Community Dentistry, School of Dentistry, Faculty of Health Science, University of Copenhagen, Copenhagen, DK-2200, Denmark; Email: lbch@sund.ku.dk; 3 Copenhagen Gerontological Oral Health Research Centre, School of Dentistry, Faculty of Health Science, University of Copenhagen, Copenhagen, DK-2200, Denmark; Email: holmp@sund.ku.dk; 4 Department of Public Health, Section of Social Medicine, and Center for Healthy Aging, University of Copenhagen, Copenhagen, DK-1014, Denmark; Email: kiav@sund.ku.dk; 5 Danish Aging Research Center, Universities of Aarhus, Southern Denmark and Copenhagen, Aarhus, DK-5000, Denmark; 6 Centre for Diabetes, Bart’s & The London School of Medicine & Dentistry, Queen Mary University of London, London, E1 2AT, UK; Email: bboucher@doctors.org.uk

**Keywords:** calcium, dairy products, elderly, oral health, periodontitis, vitamin D

## Abstract

This cross-sectional study investigates whether calcium intakes from dairy and non-dairy sources, and absolute intakes of various dairy products, are associated with periodontitis. The calcium intake (mg/day) of 135 older Danish adults was estimated by a diet history interview and divided into dairy and non-dairy calcium. Dairy food intake (g/day) was classified into four groups: milk, cheese, fermented foods and other foods. Periodontitis was defined as the number of teeth with attachment loss ≥3 mm. Intakes of total dairy calcium (Incidence-rate ratio (IRR) = 0.97; *p* = 0.021), calcium from milk (IRR = 0.97; *p* = 0.025) and fermented foods (IRR = 0.96; *p* = 0.03) were inversely and significantly associated with periodontitis after adjustment for age, gender, education, sucrose intake, alcohol consumption, smoking, physical activity, vitamin D intake, heart disease, visits to the dentist, use of dental floss and bleeding on probing, but non-dairy calcium, calcium from cheese and other types of dairy food intakes were not. Total dairy foods (IRR = 0.96; *p* = 0.003), milk (IRR = 0.96; *p* = 0.028) and fermented foods intakes (IRR = 0.97; *p* = 0.029) were associated with reduced risk of periodontitis, but cheese and other dairy foods intakes were not. These results suggest that dairy calcium, particularly from milk and fermented products, may protect against periodontitis. Prospective studies are required to confirm these findings.

## 1. Introduction

Periodontal disease is caused by a bacterial infection that induces breakdown of the connective tissue that anchors teeth to alveolar bone [1]. If left untreated, periodontal disease may lead to tooth loss. Periodontitis is among the most common chronic infections in older dentate adults, affecting 82% of 65–74-year-olds and 42% of 35–44 year-olds in a sample of 1115 Danish adults [2] and around 40% of the entire adult population in the UK [3,4]. Another study among healthy community-dwelling people over the age of 80 years found that more than half of the participants fulfilled the criteria used for severe periodontitis [5]. Prevalence of periodontitis also varies with socio-economic status [6], suggesting that lifestyle factors contribute to disease development and previous studies suggest that periodontitis risk is influenced by diet, physical activity, smoking and alcohol consumption [7,8].

A few studies have suggested that high intakes of calcium and vitamin D may enhance enamel remineralization, reduce demineralization [9], and prevent alveolar bone loss with consequent improvement in retention of the natural dentition [10]. However, attention has only recently been given to research on other possible nutritional determinants of periodontitis. Therefore, the role of non-carbohydrate nutrients in its pathogenesis remains unclear [11].

Most dairy products are good sources of calcium and their content of nutrients such as lactose and casein phosphopeptides may enhance calcium absorption and mineral retention [12]. However, the content of calcium (mg/100 g) and other components (e.g., whey, casein, bifidobacterium), may vary in different types of products [13]. When specific nutrients and their food sources are both associated with a health condition, those associations are unlikely to be spurious. Although dietary guidelines are based on nutrient contents, people eat “food”; thus, nutritional advice for health promotion and disease prevention needs to be based on foods or food patterns rather than on single nutrients. This study, therefore, aimed to investigate whether calcium intakes from dairy and non-dairy foods and the absolute intakes of these foods were associated with periodontitis, whilst allowing for differences in other lifestyle factors.

## 2. Methods

The Copenhagen Oral Health Senior Study (COHSS) is a cross-sectional study, conducted in 2004/2005, addressing issues related to aging, lifestyle factors and dental health. The study population consisted of Caucasian men and women, primarily of Scandinavian descent, aged 65 years and older who were already participants in the third follow-up of the Copenhagen City Heart Study in 2001/2003, an ongoing, longitudinal study with a focus on cardiovascular diseases within the general adult population. The baseline study conducted in 1976/1978 included 14,223 adults aged 20 years and older, living in the area of Østerbro (Copenhagen, Denmark). The participants have subsequently been examined at first follow-up in 1981/1983 (*n* = 12.698), second follow up in 1991/1994 (*n* = 10.135) and third follow up in 2001/2003 (*n* = 6.238). A detailed description of the Copenhagen City Heart Study procedures has been published elsewhere [14].

Among the subjects from the third follow-up of the Copenhagen City Heart Study who volunteered to continue in the current project, 1918 individuals, community-dwelling, aged 65 years or older identified as still living in Copenhagen were invited to participate in the present oral health study (COHSS) in 2004/2005. In total, 783 of these individuals agreed to participate (participation rate: 40%). Inclusion criteria included the ability to communicate verbally and to attend/travel to the dental clinic. Characteristics of participants (*n* = 783) and non-participants (*n* = 1135) have been fully described elsewhere [15,16]. Briefly, a larger proportion of non-participants was older and of male gender compared to participants (*p* < 0.001).

**Figure 1:**
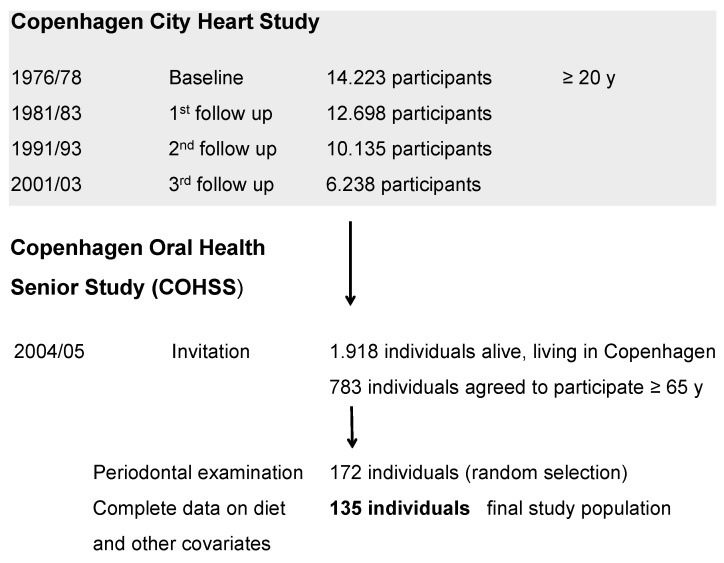
Flow chart for participants in the Copenhagen Oral Health Senior Study.

The sub-group for the present study was composed of the 135 individuals, who comprised a subset of the 783 who went through a diet history interview and had a periodontal examination, as well as complete information on all relevant covariates (Figure 1). Participants from this subset, and those not participating in the periodontal examination did not differ with respect to age, gender, education, smoking, sucrose consumption or the use of dental floss. However, the non-participants tended to have higher alcohol consumption and fewer visits to the dentist (*p* < 0.05) compared to the participants from the subset.

The data collection comprised an interview on health, psychosocial factors and oral health. The COHSS was conducted in accordance with the Helsinki Declaration and was approved by the local Ethical Committees (KF 01-144/01).

## 3. Dietary Assessment

Dietary data were collected using the “diet history interview”, which forms the basis for the analyses of the habitual intake of foods, nutrients, dietary patterns and habits. The diet history interviews were used to estimate average dietary intakes based on information about diet in the past month. Participants were questioned about their meal patterns and contents determined by interview using pre-coded questions, while quantitative information on food intake and meal and portion sizes was collected using photo-series, cups and measures. After the usual pattern of eating had been described, a checklist of the frequency of consumption of specific foods was administered. The structured diet history interview was validated for use in the older populations [17]. All interviews were performed by a single dietician. In total, 734 participants (out of 783) provided complete information on diet. 

Total dietary calcium intake (mg/day) was estimated using the Dankost program [18] and stratified by food source for calcium, *i.e.*, calcium from dairy sources and calcium from non-dairy sources. Dairy calcium intake was calculated from all dairy foods as well as dairy ingredients from recipes for other foods, for example, cheese from pizza and milk from mashed potatoes. Non-dairy calcium was calculated by subtracting dairy calcium from total dietary calcium intake, including supplemental calcium intake. Due to limited information on calcium dosage of supplements used, 800 mg was added to the total amount of calcium intake, but only for those who reported taking calcium supplements daily. Calcium intakes from tap or bottled water were also accounted for. 

Total dairy food intake (g/day) was classified into four groups: milk (whole and skimmed milk), cheese (hard and soft cheese), fermented foods (yogurt and lactic acid drinks) and other foods (butter, ice-cream, creams, *etc.*).

## 4. Oral Examination

One dentist (KH), carefully trained by an experienced clinical examiner (PH-P), performed all clinical oral examinations. The examination included assessment of caries, restoration, number of teeth, presence of visible detectable plaque registered at six sites per tooth in all participants. Standard dental equipment was used and no radiographs were taken [15].

In a randomly selected subset of participants with full or partial dentition (*n* = 172), full-mouth periodontal pocket depths, bleeding on probing, the presence of furcation involvements and of tooth mobility were recorded. Pocket depths were measured with a manual periodontal probe at six sites per tooth for all teeth present. Clinical attachment loss is the distance from the cement-enamel junction to the bottom of the pocket. The distance from the free gingival margin to the cement-enamel junction was measured and clinical attachment loss was calculated by subtracting this value from pocket depth [19,20]. Gingival recession was recorded as a negative value which was then added to pocket depth. For each individual, bleeding scores were determined as the percentage of sites with bleeding relative to the total number of sites examined. Periodontitis was defined as number of teeth with attachment loss of 3 mm or greater (count data).

## 5. Covariates

The socio-demographic variables were age (continuous variable), gender and educational level, classified as ≤7 years *vs*. >7 years of education (representing completed elementary school attendance).

Lifestyle characteristics included physical activity, smoking and intakes of alcohol and sucrose. Individuals were classified as sedentary if they reported no leisure time activity or light activity less than twice a week. In this study, only subjects who smoked daily were considered current smokers (yes/no). Alcohol consumption was classified as above *vs.* within recommendations according to the Danish National Board of Health: maximum consumption of 168 g alcohol/week for women and 252 g alcohol/week for men [21]. Sucrose consumption was classified as ≤10% *vs.* >10% of total energy intake, in accordance with the suggested threshold limit for potential increases in caries rates, and also associated with lower consumption of vitamins and minerals [22].

Activated vitamin D (1,25(OH)2D) plays a role in maintaining oral health through its effects on calcium and bone metabolism and innate immunity. Total intake of vitamin D2 and D3 (μg/day) was estimated using dietary sources and, if supplementation was taken daily, as 10 μg for vitamin D supplement use, 5 μg for multivitamin supplement use, and 6 μg for daily use of cod liver oil. Only 4% of participants reported vitamin D intakes within current recommendations (15 μg/day for adults aged 51–70 years and 20 μg/day for those aged ≥71 years) [23]. Vitamin D intakes were, therefore, classified as within *vs.* above the 50th percentile (6.8 μg/day).

Supplementary calcium is currently being *queried* as increasing risks of heart disease, whilst dietary calcium does not [24]. Researchers have found that individuals with periodontitis are more likely to suffer from coronary artery disease than those without periodontitis [25]. Therefore, self-reported coronary heart disease (yes/no) was considered in this study. 

The majority of the study population (98%) reported daily tooth brushing and, regular use of dental floss (yes/no) was, therefore, used as a measure of oral-health-related behavior instead. Elapsed time since last dental care visit (≤12 *vs.* >12 months), number of remaining teeth, and bleeding scores were also included in the models. Bleeding on probing was categorized based on the 75th percentile (<30% *vs.* ≥30%).

## 6. Statistical Analysis

Characteristics of the study population were presented as “mean (SD)” or “percentage (*n*)”. The outcome for analyses was the number of teeth with periodontal attachment loss, modeled as “count” data. Poisson regression, adjusted for potential confounders, was used to evaluate associations between intakes of calcium and dairy foods and periodontitis. The estimated coefficients were expressed as incidence-rate ratios (IRR), that is, exponential of Poisson coefficient. IRR are presented for each 100 mg daily increase in calcium intake (which corresponds to approximately 100 mL of milk or yogurt) and 100 g daily increase in dairy foods intake. Effect modification by gender was evaluated for interaction, but as no significant difference between genders was found, data analyses were not stratified by gender. The level of significance was set at *p* < 0.05. All statistical analyses were performed using the STATATM software (Stata Corp 9.2, TX, USA).

## 7. Results

The study population was composed of 53% women and the age ranged from 66.6 to 95.5 years (mean 76.3 years). Total dietary calcium intake, excluding daily supplementation, ranged from 309 mg to 3373 mg (mean 1011 mg). Only 5% of the population reported daily intake of calcium supplements. Dairy foods were the major source of dietary calcium, accounting for 56% of total calcium intake. The mean calcium intakes from milk, cheese and fermented foods were 223 mg, 217 mg and 121 mg, respectively. The mean intake from dairy foods was 334 g/day (range: 34–2247 g). Milk was the major source of dairy consumption (185 g) followed by fermented foods (98 g), cheese (36 g) and other types dairy food (15 g). 

The mean number of remaining teeth in the oral cavity, and of teeth with periodontal attachment loss were 20 and 6.3, respectively. The mean pocket depth and attachment loss were 2.0 mm and 3.4 mm, respectively. Further characteristics of the study population are presented in Table 1.

**Table 1:** General characteristics of the study population.

Characteristics	% (*n*)
Educational level ≤7 years	31.8 (43)
Sedentary	20 (27)
Current smoking	26 (35)
Vitamin D intake <6.8 μg/day	49.6 (67)
>10% energy from sucrose	15 (20)
Alcohol above recommendations	36.3 (49)
Heart disease	49.6 (67)
>12 months since last dental care visit	7.4 (10)
No regular use of dental floss	86 (116)
Bleeding score ≥30%	24.0 (32)

Table 2 shows the incidence risk ratio (IRR) of having teeth with periodontal attachment loss per 100 mg increase in intake of calcium (mg/day) from dairy and non-dairy sources. Intakes of total dairy calcium and calcium from milk were inversely and significantly associated with risk of periodontitis in both crude and adjusted models. In the univariate analysis, intake of calcium from fermented foods was not significantly associated with periodontitis (IRR = 0.99; *p* = 0.667), but this association became significant after adjustment for covariates (IRR = 0.96; *p* = 0.030), particularly after inclusion of oral health-related variables, such as: visits to the dentist, use of dental floss and bleeding on probing. Intakes of calcium from cheese or from non-dairy sources were not significantly associated with periodontitis regardless of adjustment for covariates.

Table 3 shows the IRR of having teeth with periodontal attachment loss per 100 g increase in intake of dairy foods (g/day). The associations for dairy food intakes followed the same pattern of calcium intake. Intakes of all types of dairy food and milk were inversely and significantly associated with risk of periodontitis in both crude and adjusted models. Intake of fermented foods was not significantly associated with periodontitis in the crude model (IRR = 0.99; *p* = 0.720), but the association became significant after adjustment for covariates (IRR = 0.97; *p* = 0.029). Cheese intake was not significantly associated with periodontitis in either crude or adjusted models.

**Table 2:** The association (IRR *) between dairy and non-dairy calcium intakes and number of teeth with attachment loss ≥ 3 mm.

	Dairy Calcium (mg/day)				Non-Dairy Calcium (mg/day)				
	Crude		Adjusted ^†^		Crude			Adjusted ^†^	
	IRR *	*p*	IRR *	*p*	IRR *	*p*	IRR *		*p*
	(95% CI)		(95% CI)		(95% CI)		(95% CI)		
Total	0.98	0.008	0.97	0.021	0.98	0.097	0.99		0.340
	(0.95–0.99)		(0.96–0.99)		(0.96–1.00)		(0.96–1.02)		
Milk	0.96	0.001	0.97	0.025					
	(0.93–0.98)		(0.95–0.99)						
Cheese	0.99	0.892	0.99	0.701					
	(0.97–1.02)		(0.96–1.03)						
Fermented foods	0.99	0.667	0.96	0.030					
	(0.95–1.03)		(0.92–0.99)						

* Incidence-rate ratio (IRR) is for each 100 mg daily increase in calcium intake; ^†^ Model adjusted for age, gender, education, sucrose intake, alcohol consumption, smoking, physical activity, vitamin D intake, heart disease visit to the dentist, use of dental floss , number of remaining teeth and bleeding on probing.

**Table 3:** The association (IRR*) between intake of dairy foods and number of teeth with attachment loss ≥3mm.

	Dairy Foods (g/day)			
	Crude		Adjusted ^†^	
	IRR * (95% CI)	*p*	IRR * (95% CI)	*p*
Total	0.96 (0.93–0.98)	0.002	0.96 (0.96–0.99)	0.003
Milk	0.95 (0.93–0.99)	0.001	0.96 (0.93–0.99)	0.028
Cheese	0.98 (0.97–1.02)	0.821	0.95 (0.78–1.16)	0.625
Fermented foods	0.99 (0.94–1.04)	0.720	0.97 (0.95–0.99)	0.029

* Incidence-rate ratio (IRR) is for each 100 g daily increase in intake of dairy foods; ^†^ Model adjusted for age, gender, education, sucrose intake, alcohol consumption, smoking, physical activity, vitamin D intake, heart disease, visit to the dentist, use of dental floss, number of remaining teeth and bleeding on probing.

Neither the absolute intake (g/day) of other types of dairy foods (e.g., butter, ice-cream, cream), nor calcium intakes from other types of dairy foods were significantly associated with periodontitis (results not shown).

## 8. Discussion

Our findings showed that intakes of both calcium and dairy foods, in particular milk and fermented products, were significantly and inversely associated with periodontitis. Intakes of non-dairy calcium, calcium from cheese, and other dairy products were not associated with periodontitis. These findings are in line with some earlier studies [26,27].

Two studies have investigated the relationship between intakes of dairy foods and periodontitis [26,27], but only one has stratified the analysis according to type of dairy foods [27]. Al-Azhrani found that intakes of all dairy foods was inversely and significantly associated with periodontitis, defined as pocket depth ≥4 mm and attachment loss ≥3 mm [26]. Shimazaki *et al.* found that an increased intake of lactic acid/fermented foods was associated significantly with lesser mean pocket depth and attachment loss, whereas no significant associations were found with intakes of cheese, milk and other dairy foods [27]. Due to the fact that the amount of calcium in milk is almost the same as that in yogurt, it was argued that calcium from dairy foods may not have a meaningful impact on periodontitis, possibly because probiotic bacteria (e.g., *Lactobacillus* and *Bifid* bacterium) from fermented/lactic acid rich foods may provide protective effects for periodontitis by suppressing the growth of periodontal pathogens in the oral cavity and stimulating the immune system [28]. However, neither of the two previous studies quoted considered the potential for independent effects of calcium and hence it is not possible to infer that calcium intakes had no effect on periodontitis.

In the present study, the magnitude of the associations between intakes of either milk (IRR = 0.96) or fermented foods (IRR = 0.93) with periodontitis did not differ (*p* > 0.05). Milk intake was consistently associated inversely with periodontitis in both crude and adjusted models, while cheese intake was not. However, we cannot exclude the possibility that this lack of association might be due to inadequate statistical power attributable to the low range of cheese intakes (0 g to 354 g) compared to those of milk (0 g to 2197 g) or of fermented foods (0 g to 1000 g).

The assessment of periodontal disease varies markedly between studies as there is no generally approved case definition for use in population-based surveillance of periodontitis [20]. In the present study, high-standard assessments included full mouth examination (4 quadrants), and of 6 sites per remaining tooth, by an experienced and serially calibrated dentist. Since periodontitis is very selective, *i.e.*, severe periodontitis may affect few teeth and sites in many individuals, overall mean measures may underestimate the true prevalence of serious periodontitis [20]. We therefore used the number of teeth with attachment loss ≥3 mm at any site, though underestimation of our outcome variable may mean that there is a stronger association between dairy and calcium intake and periodontitis than we have estimated since our results may provide under rather than over estimates of association. In order to perform sensitivity analysis regarding our case definition, the threshold for periodontal attachment loss was set at 6 mm and 8 mm, respectively for moderate and severe periodontitis. The analyses showed the same pattern of results, but some associations did not reach statistical significance.

Strengths of this study were that we could adjust analyses for variations in a wide range of risk-factors for periodontitis; calculated dietary intakes were based on dietary history interviews using food models and photographs to optimize estimations of portion size. The main advantage of this method is that long-term dietary habits can be estimated with relatively high validity [29]. However, the possibility of inaccurate estimates of calcium and dairy products consumption cannot be completely excluded due to the fact that the habitual dietary intake assessments were based on self-reported intakes. 

A number of limitations should be noted. Only, a randomly selected subset of the study population underwent periodontal examinations. Exclusions due to insufficient information on dietary intake, clinical periodontal examination or other covariates, limited the number of participants eligible for analysis. However, completers and non-completers were comparable for the main socio-demographic characteristics, though non-completers had somewhat higher alcohol consumption and fewer visits to the dentist than participants. Thus, responders tended to have lower-risks, leading to attenuation rather than inflation of the observed associations, suggesting that our findings have increased, rather than reduced validity. Another potential limitation was the lack of detailed quantitation of supplemental calcium intakes. Thus, since we only allowed for calcium supplementation in daily users, we may have underestimated intakes of non-dairy calcium which may have attenuated, rather than inflated, our findings. However, since we found calcium from non-dairy sources had no appreciable association with risk of periodontitis, calcium from non-dairy sources may have low bioavailability, and thus no effect on oral health. The absorption of dietary calcium is a critical factor in determining the availability of calcium for bone mineralization [30]. Healthy individuals absorb about 20%–35% of the calcium in dairy foods and ingredients [31], while calcium absorption from calcium fortified non-dairy foods (such as fortified soy milk) is 25% less than that from dairy sources [32]. Although these results are consistent with our previous study showing that non-dairy calcium was not significantly associated with tooth loss [33], we cannot exclude the possibility that the lack of association might be due the relatively lower range of non-dairy calcium intake (75 mg to 1620 mg) compared to total dairy calcium intake. 

In addition to dietary calcium, vitamin D status is an important factor influencing intestinal calcium absorption [12]. We found no specific effects for vitamin D, nor any interactions between vitamin D and calcium intakes in the present study, however, due to the limited sample size it was not possible to stratify analyses by vitamin D intake and this possible interaction requires further investigation.

Finally, given the obviously chronic nature of periodontitis, diet several years earlier may be responsible for the current oral health status. Reduced masticatory ability caused by tooth loss, use of dentures, or loose teeth may have led to changes in dietary choices with increased consumption of dairy foods because they are the easiest to chew [34]. If this was the case in our study, this would have led to attenuation rather than inflation of the observed associations. However, as we had no information on previous dairy consumption we could not assess this possibility. Therefore, future prospective studies should document serial dairy consumption.

## 9. Conclusion

The present cross-sectional study suggests that higher daily intakes of milk and fermented foods may be protective against periodontitis. In addition, higher intakes of calcium from dairy products, particularly from milk and fermented foods, were inversely associated with severity of periodontal attachment loss. However, further prospective studies are needed to investigate the relationships between serial intakes of dairy foods and their calcium content, and of vitamin D status, with development of periodontitis for evidence of causality.
